# Calibration of Self-Reported Time Spent Sitting, Standing and Walking among Office Workers: A Compositional Data Analysis

**DOI:** 10.3390/ijerph16173111

**Published:** 2019-08-27

**Authors:** David M. Hallman, Svend Erik Mathiassen, Allard J. van der Beek, Jennie A. Jackson, Pieter Coenen

**Affiliations:** 1 Centre for Musculoskeletal Research, Department of Occupational Health Sciences and Psychology, University of Gävle, 80637 Gävle, Sweden; 2 Department of Public and Occupational Health, Amsterdam UMC, Vrije Universiteit Amsterdam, Amsterdam Public Health Research Institute, 1081 BT Amsterdam, The Netherlands

**Keywords:** physical activity, sedentary behavior, office work, accuracy, calibration, compositional data analysis

## Abstract

We developed and evaluated calibration models predicting objectively measured sitting, standing and walking time from self-reported data using a compositional data analysis (CoDA) approach. A total of 98 office workers (48 women) at the Swedish Transport Administration participated. At baseline and three-months follow-up, time spent sitting, standing and walking at work was assessed for five working days using a thigh-worn accelerometer (Actigraph), as well as by self-report (IPAQ). Individual compositions of time spent in the three behaviors were expressed by isometric log-ratios (ILR). Calibration models predicting objectively measured ILRs from self-reported ILRs were constructed using baseline data, and then validated using follow-up data. Un-calibrated self-reports were inaccurate; root-mean-square (RMS) errors of ILRs for sitting, standing and walking were 1.21, 1.24 and 1.03, respectively. Calibration reduced these errors to 36% (sitting), 40% (standing), and 24% (walking) of those prior to calibration. Calibration models remained effective for follow-up data, reducing RMS errors to 33% (sitting), 51% (standing), and 31% (walking). Thus, compositional calibration models were effective in reducing errors in self-reported physical behaviors during office work. Calibration of self-reports may present a cost-effective method for obtaining physical behavior data with satisfying accuracy in large-scale cohort and intervention studies.

## 1. Introduction

Excessive time spent sitting and standing have both received considerable attention as possible risk factors for musculoskeletal disorders [1,2], cardiovascular diseases [3,4,5] and early mortality [6]. Incorporating moderate to vigorous physical activity, such as walking, in daily routines shows numerous health benefits [7,8] and may counteract some of the detrimental health effects of prolonged sitting [9]. However, evidence has predominantly been obtained from studies of leisure-time behaviors. There is a growing interest in understanding how physical behaviors at work (including sitting, standing and walking) contribute to overall physical activity and health [10,11]; however, epidemiological studies investigating these associations remain inconclusive [10,12,13].

Studies devoted to physical behaviors and health have often relied on self-reported behavioral estimates. However, self-reported estimates have been shown to disagree with objective measurements, for example, accelerometry [14,15,16,17,18]. Inaccuracy (as defined by the International Organization for Standardization [19]) can result in misleading dose–response associations with health outcomes, thus hampering the development of effective recommendations and guidelines on physical behaviors during work and leisure. Objective methods offer more accurate information on time use in different physical behaviors than self-reports [20]. Some studies have assessed physical behaviors among office workers using, for example, accelerometry [21], but they are, in general quite small, likely because collecting and processing data are associated with substantial costs [22,23,24]. Questionnaires are considerably more affordable and more feasible in large-scale epidemiological studies, and it is therefore of interest to assess the extent to which self-reported data offer information that can be used to predict objectively measured ‘true’ physical behaviors.

Calibration of self-reported estimates of physical behaviors by regressing self-reported data against objectively measured data has been shown to improve data accuracy [15,16,25,26]. While several studies have developed such calibration models, only a few addressed physical behaviors at work, and they focused uniquely on blue-collar workers [15,16]. Physical behavior patterns in blue-collar workers who spend a large proportion of working time standing and walking will likely differ markedly from those in office workers who mainly sit and stand at work [21,27,28]. Thus, previous calibration models for blue-collar workers may have limited relevance to calibration of self-reported physical behaviors during office work.

Previous studies that calibrated self-reports focused on an isolated physical behavior such as sitting time [15], without considering how time is distributed across multiple behaviors during a complete working day. This is problematic since time is inherently constrained and all component behaviors must add up to a whole; for example, 24 h in a day or 8 h at work. This constrained, closed property of data is described by the term “compositional” [29,30]. As a result of this compositional structure, spending more time in one behavior (e.g., sitting) will inherently result in less time spent in at least one of the other possible behaviors (e.g., standing or walking). This co-dependency entails that traditional statistics may produce misleading results if single behaviors are examined without consideration to other behaviors in the composition [29,31]. Compositional data analysis (CoDA), which has recently been introduced in physical activity research, offers a set of statistical procedures for effectively dealing with compositional data [29,32]. CoDA is based on the premise that the information contained in a composition is better expressed in terms of ratios between the parts than by stating each part separately [33]. Ratios, however, can take only positive values, and in order to arrive at data in an unconstrained real space, CoDA requires the ratios to be log-transformed, preferably using so-called isometric log-ratio transformation [34]. The resulting data can be analyzed using standard statistical methods, since they are no longer constrained and closed [29,30].

We have recently shown that calibration within a CoDA framework can be effective in improving the accuracy of self-reported time in a single behavior (sitting) among office workers [35]. It is likely that calibration of a particular behavior will be even more effective, that is, lead to more accurate estimates of ‘true’ behavior, if self-reports of all other component behaviors are also included as predictors. To our knowledge, no study has evaluated the efficacy of calibration models in a CoDA framework to predict a complete set of physical behaviors, including sitting, standing and walking.

The aim of this study was to develop and evaluate, within a CoDA framework, calibration models predicting objectively measured time spent sitting, standing and walking among office workers from the composition of self-reported behaviors, and to validate the models using new data.

## 2. Materials and Methods

### 2.1. Design and Study Sample

Data from a controlled intervention study on relocation from traditional (cell or open plan offices) to activity-based offices were used. Design, population and data collection of the study have been described in detail elsewhere [36,37]. In brief, the study was conducted at a large Swedish governmental agency (the Swedish Transport Administration) between May 2015 and January 2017. Data were collected before office relocation (baseline) and 3 and 12 months after relocation (follow-up). In the present study, we developed calibration models using baseline data, which were then validated using 3-month follow-up data.

We recruited office workers from five office sites, located across Sweden. Inclusion required current employment at any of these office sites. Workers were excluded if they were on sick leave or parental leave, did not move to one of the new offices (applicable only for the intervention group), or retired or changed job during the study time.

A total of 901 office workers were invited to participate in a web-based questionnaire administered by e-mail at baseline. Of the invited workers, 493 responded to the baseline questionnaire and 117 indicated interest in participating in objective measurements of physical behaviors. Baseline measurements for both self-reported and objectively measured data were obtained from 98 workers, and viable self-reported and objective data were obtained from 73 of these workers at 3-months follow-up.

The study was approved by the Regional Ethical Review Board in Uppsala, Sweden (registration no. 2015/118) and all participants provided written informed consent prior to participation.

### 2.2. Measurements

At baseline and follow-up, participants filled out a comprehensive web-based questionnaire, physical behaviors were measured objectively during one full week and participants recorded daily working hours in a diary. The questionnaire was administered either shortly before or shortly after taking part in the objective measurement protocol (see below).

### 2.3. Demographic Information

Self-reported information on age (years), gender (man or woman), educational level (public school, high school, vocational or university), position (manager or employee), office type (private or shared room/open-plan office), full-time employment (yes or no), and seniority in the work tasks (years) and organization (years) were collected using the baseline questionnaire.

### 2.4. Self-Reported Time in Sitting, Standing and Walking

Self-reported data on time spent sitting, standing and walking at work were collected using the International Physical Activity Questionnaire (IPAQ) [38]. The question on sitting, that is, “*During the past 7 days, how much time did you spend sitting during a typical workday?*” was adapted also to walking and standing, and responses were provided in hours and minutes per day.

### 2.5. Objectively Measured Time Use

Physical behaviors including sitting, standing and walking were objectively recorded using a tri-axial accelerometer (Actigraph GTX+, ActiGraph LLC, Pensacola, FL, USA) attached to the thigh according to previously described procedures [20]. Data were sampled at 30 Hz and off-line processed using the customized Acti4 Software (National Research Centre for the Working Environment, Copenhagen, Denmark). This software can identify uninterrupted periods of sitting, standing and walking with high sensitivity and specificity [20,39]. The time-line of these activities was synchronized with diary records of working hours, visually inspected and processed using Spike2 (version 7.03, Cambridge Electronic Design, Cambridge, UK). Non-wear periods and technical errors were excluded based on diary notes, visual inspection and an automatic algorithm identifying periods greater than 4 h with no change in body position. For each worker, the number of minutes spent sitting, standing and walking at work was determined for each day and then averaged across days. We also detected other physical behaviors, such as running and cycling. However, as these behaviors were extremely rare during office work, they were excluded from further analyses (mean (SD) time running 0.1 (0.2) min/day and of cycling 1.3 (3.0) min/day).

### 2.6. Compositional Expressions of Physical Behaviors

Time spent in different physical behaviors is compositional, and thus conventional statistical analyses should be used only after converting time in each individual behavior to a log-ratio of time in that behavior relative to time spent in the other behavior(s) [29,30]. Since log-ratios cannot be computed if the numerator or the denominator is zero, we replaced any zeroes in self-reported minutes of standing (*n* = 8) and walking (*n* = 14) by 1 min. Similar replacement procedures have been applied in previous CoDA studies to handle zeroes [32]. The current approach was considered reasonable since office workers rarely engage in less than one minute of standing or walking during a normal working day, as confirmed by the objective measurements in the present study. No zeroes occurred for self-reported sitting or for any of the objectively measured behaviors.

Following the procedure proposed by Dumuid et al. [30], we calculated three sets of isometric log-ratios (ILRs), rotating the sequence of behaviors so that each behavior (e.g., sitting) was prioritized as the first compositional part in a set, and the ratio between the two remaining behaviors (e.g., stand/walk) occurred as the second compositional part. Thus, each set expressed a composition of all three behaviors; sitting, standing and walking. The following equations were used to calculate these three sets of ILRs, prioritizing sitting, standing and walking as follows:

(i) sitting:

$$ILR1_{Sit/nonsit}=\sqrt{\frac{2}{3}}\ln\left(\frac{Sit}{\sqrt[{2}]{Stand\times Walk}}\right)$$

$$ILR2_{Stand/walk}=\sqrt{\frac{1}{2}}\ln\left(\frac{Stand}{Walk}\right)$$

(ii) standing:

$$ILR1_{Stand/nonstand}=\sqrt{\frac{2}{3}}\ln\left(\frac{Stand}{\sqrt[{2}]{Sit\times Walk}}\right)$$

$$ILR2_{Sit/walk}=\sqrt{\frac{1}{2}}\ln\left(\frac{Sit}{Walk}\right)$$

and (iii) walking:

$$ILR1_{Walk/nonwalk}=\sqrt{\frac{2}{3}}\ln\left(\frac{Walk}{\sqrt[{2}]{Sit\times Stand}}\right)$$

$$ILR2_{Sit/stand}=\sqrt{\frac{1}{2}}\ln\left(\frac{Sit}{Stand}\right)$$

The transformation procedure is summarized in Supplementary Material File S1, step A.

### 2.7. Statistical Analyses

All statistical analyses were conducted using SPSS version 24 (IBM, Armonk, NY, USA). Descriptive metrics are presented as means with standard deviation (SD) for continuous data and as counts (% of total) for categorical data.

Calibration models expressing ‘true’ ILRs obtained from objectively measured time spent in sitting, standing and walking from ILRs obtained from self-reported time spent in these behaviors were constructed using linear regression analysis on baseline data.

Three linear regression models were constructed on ILR data, one for each of the three sets of ILRs, as defined in the equations above. In these models, both self-reported ILR coordinates pertaining to a modelled behavior (e.g., ILR1, sit/nonsit and ILR2, stand/walk) and their interaction (ILR1 × ILR2) were included as predictors, and the corresponding first coordinate (e.g., ILR1, sit/nonsit) of the objectively measured behavior was used as the outcome. Thus, we accounted for the complete self-reported composition of behaviors when predicting ‘true’ time use, in terms of ILR1, for each prioritized behavior. The calibration model procedure is summarized in Supplementary Material File S1, step B.

For each model, we derived the intercept and the regression coefficients (B) with standard errors (SE) and *p*-values. The overall ability of the calibration model to explain variance in ‘true’ exposure was evaluated by the R square (R^2^) quotient. The efficacy of the model in improving accuracy of self-reports was evaluated by the root-mean-square error (RMS), that is, the RMS difference between self-reported and objectively measured ILRs, and was further expressed as a percentage of the RMS error of un-calibrated data. The distribution of the residuals in each model was inspected using histograms, and no marked deviations from normality were observed.

Calibration models were validated by applying the regression equations obtained on baseline data to the data obtained at 3-months follow-up and assessing model efficacy in terms of RMS error as explained above.

While the prioritized ILR1 was the major focus for calibration in each of the three sets of ILR coordinates, we also developed regression models predicting the ‘true’ ILR2 from self-reported ILR1 and ILR2 in each of the sets. These models were not validated. Calibration of ILR2 is included in the procedure summarized in Supplementary Material File S1, step B.

## 3. Results

### 3.1. Study Sample

The study sample, that is, workers both responding to the questionnaire and taking part in objective measurements, comprised 98 office workers, approximately half men and half women, with a mean age of 47 years, a mean seniority in the organization of 13 years, and most having full-time employment (Table 1). Most of the participants had a university degree. A total of 59% and 41% worked in private offices and shared room/open-plan offices, respectively.

The study sample was comparable in most respects to workers only responding to the questionnaire (*n* = 395), even though the study sample showed a slightly larger proportion of women and workers with full-time employment (results not shown).

### 3.2. Self-Reported and Objectively Measured Physical Behavior at Work

At the group level, the objective measurements showed that most of the working time was spent sitting (70%), some time was spent standing (24%), and little time was spent walking (6%) (Table 2). These group means showed high agreement with group means calculated from self-reported data, which slightly overestimated time spent sitting (by 2% of the working day) and walking (by 1% of the day), and underestimated time standing (by 2% of the day). At the level of individual workers, however, self-reports were, on average, associated with considerable inaccuracy, with RMS errors for self-reported sitting, standing and walking time being 16%time, 14%time and 7%time, respectively.

**Figure 1:**
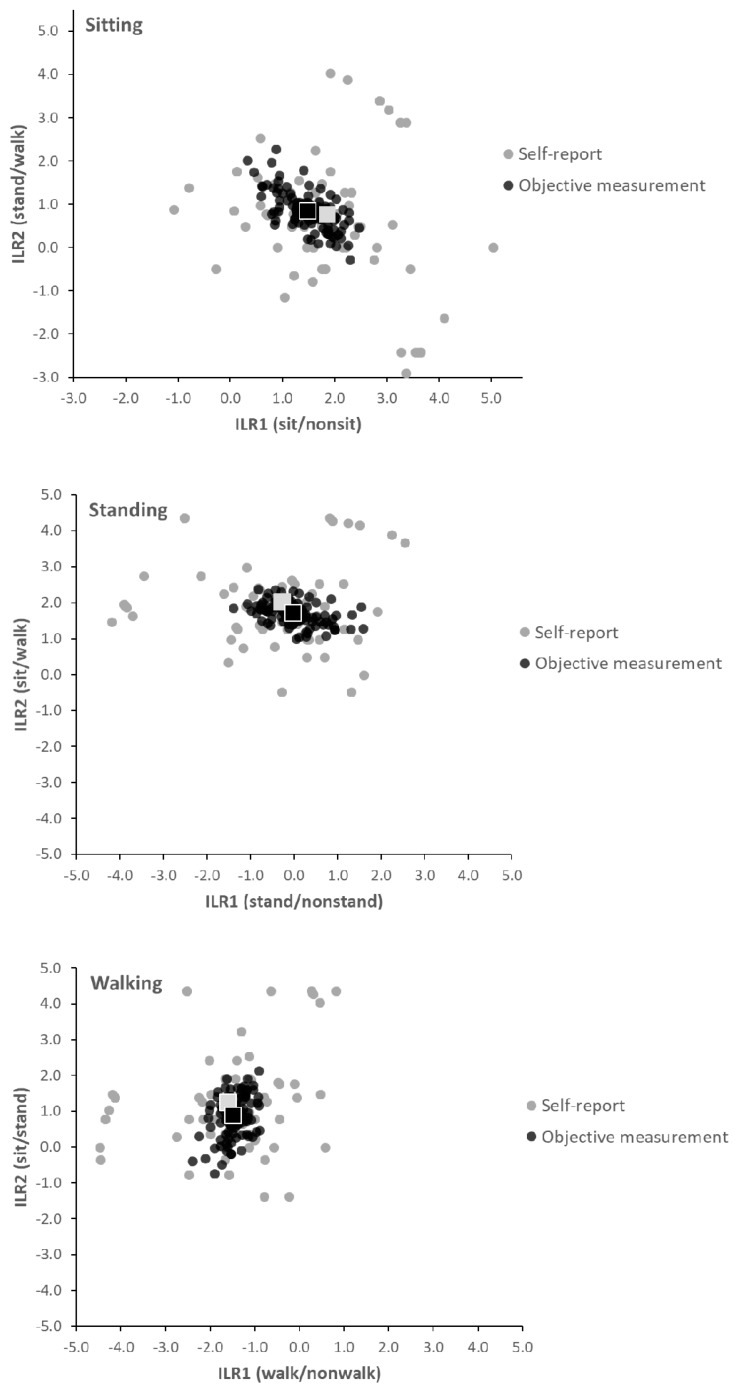
Distribution in the population (*n* = 98) of self-reported (grey circles) and objectively measured (black circles) physical behaviors (sit, stand, walk), expressed in terms of two isometric log-ratios (ILR1 and ILR2). The first ILR (*x*-axis) expresses time spent in one behavior, as stated in the upper left corner of each diagram, relative to time in the two remaining behaviors (e.g., sit/nonsit), and the second ILR (*y*-axis) expresses the relative time spent in each of these two behaviors (e.g., stand/walk when sit/nonsit is the first ILR). Squares show group means for self-report (grey) and objective measurement (black).

Self-reported and objectively measured time use at the individual level are illustrated in Figure 1, showing data in terms of ILR1 and ILR2 for each compositional set of behaviors (see Section 2.6. Compositional Expressions, above). For all behaviors (sitting, standing and walking), the dispersion in data was larger for self-reported ILRs compared to objective measurements, while mean values agreed reasonably well.

### 3.3. Calibration of Self-Reported Physical Behaviors at Work

**Figure 2:**
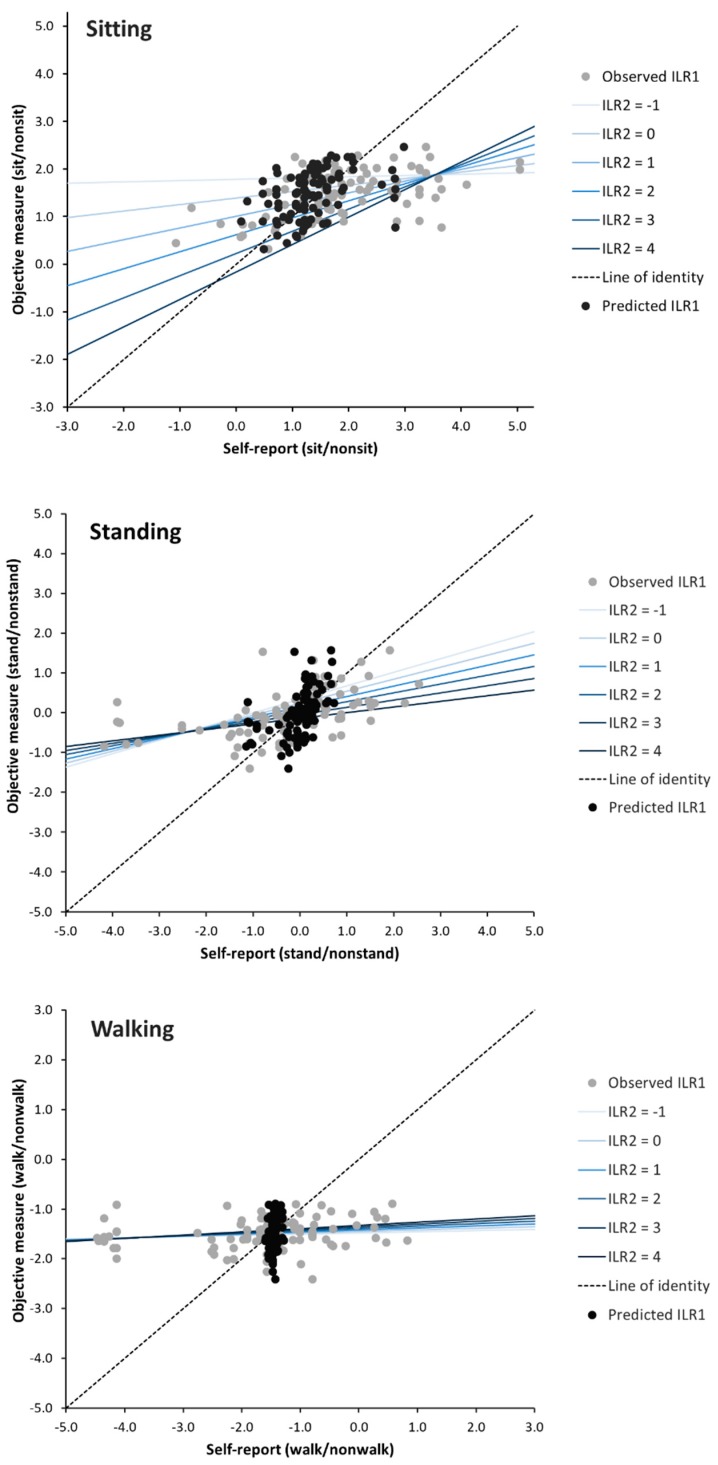
Self-reported and objectively measured time spent sitting, standing and walking. The *x*-axis represents ILR1 (e.g., sit/nonsit in the “sitting” diagram) obtained from self-reports before (grey circles) and after (black circles) calibration. The *y*-axis represents the same ILR calculated from objective measurements, that is, the ‘true’ ILR. As illustrated, calibration moves estimates closer to the ‘truth’, that is, closer to the line of identity. Colored lines illustrate the calibration model (see Table 3) at different values of ILR2 (e.g., stand/walk in the “sitting” diagram).

Un-calibrated self-reports expressed in terms of ILRs showed RMS errors of 1.03, 1.24 and 1.21 for sitting, standing and walking, respectively (Table 3, RMS before calibration). Calibration reduced these errors to 36% (sitting), 40% (standing) and 24% (walking) of the RMS error associated with un-calibrated ILRs (Table 3). Thus, calibration considerably improved the accuracy of self-reported compositions of sitting, standing and walking, as is also illustrated in Figure 2.

The R^2^ of the calibration models (Table 3) showed that the composition of self-reported time use contributed moderately to the variance in ‘true’ sitting (39%) and standing (27%), and that explained variance was even less for the model predicting walking (5%). Thus, self-reported walking contained limited information about ‘true’ walking.

When predicting ‘true’ sitting, we found that all four model parameters, that is, intercept, self-reported ILR1 (sit/nonsit) and ILR2 (stand/walk), and their interaction (ILR1 × ILR2), contributed significantly to the calibration (Table 3). The overall model predicted that increasing time in standing relative to walking (i.e., an increase in ILR2) was associated with both reduced sitting (main effect) and a stronger positive relation between self-reported and true sitting (interaction). This is also apparent from the distinctly differing prediction curves for different values of ILR2 (blue lines) in Figure 2.

The second ILR, that is, ILR2, contributed less to prediction of standing and walking, with non-significant B estimates (Table 3). The limited importance of ILR2 in these cases is also illustrated by the closeness of the prediction curves for standing and, particularly, walking at different values of ILR2 (Figure 2).

### 3.4. Evaluation of the Calibration Models at Three-Months Follow-Up

At follow-up, the RMS errors for the self-reported ILR1 in sitting, standing and walking were 1.14, 1.14 and 0.93, respectively. Calibration using the regression equation developed on the baseline data (Table 3) reduced these errors to 0.37 (sitting), 0.58 (standing) and 0.29 (walking), corresponding to 33%, 51% and 31% of the RMS error associated with un-calibrated ILR1 data. Thus, the calibration models were approximately equally effective at follow-up as at baseline.

### 3.5. Calibration Example

Using the equations above describing ILR transformations and the calibration models in Table 3, self-reported behaviors can be calibrated for any composition of sitting, standing and walking. Consider, for example, an office worker reporting sitting, standing and walking at work for 60%time, 25%time, and 15%time, respectively. Transformed into ILR coordinates, this corresponds to self-reported *ILR*1*_sit/nonsit_* = $\sqrt{\frac{2}{3}}\ln\left(\frac{60}{\sqrt[{2}]{25\times 15}}\right)$ = 0.92, and *ILR*2*_stand/walk_* = $\sqrt{\frac{1}{2}}\ln\left(\frac{25}{15}\right)$ = 0.36, when sitting is the prioritized behavior. With standing and walking as prioritized behaviors, the ILR coordinates (ILR1, ILR2) are (−0.15, 0.98) and (−0.77, 0.62), respectively. Using the “sitting” regression coefficients in Table 3, the ‘true’ *ILR*1*_sit/nonsit_* is then predicted to be ILR1 = 1.39 + 0.14 × 0.92 − 0.39 × 0.36 + 0.11 × 0.92 × 0.36 = 1.42. This result can also be obtained by reading the “sitting” diagram in Figure 2 at an x-value of 0.92, noting that an ILR2 = 0.36 falls in-between the lines illustrating ILR2 = 0 and ILR2 = 1. Corresponding calculations with standing and walking as the prioritized behaviors give self-reported *ILR*1*_stand/nonstand_* and *ILR*1*_walk/nonwalk_* of −0.15 and −0.77, respectively. Using the regression parameters in Table 3 (standing, walking), the ‘true’ *ILR*1*_stand/nonstand_* and *ILR*1*_walk/nonwalk_* are predicted to be 0.11 and −1.46, respectively. Transformation and calibration procedures are both summarized in Supplementary Material File S1, steps A and B.

While we emphasize that further use of calibrated behaviors, for instance in epidemiologic studies, should be based on ILR-transformed data, it may be of interest to examine these calibrated behaviors even in real space. In the example above, calibration of sitting resulted in a predicted ‘true’ *ILR*1*_sit/nonsit_* of 1.42. Back-transforming this calibrated ILR1 to %time also requires the predicted ‘true’ *ILR*2*_stand/walk_* (Supplementary Material File S1, step C). Using the regression parameters for “sitting” reported in Supplementary Material File S2 gives a predicted ‘true’ *ILR*2*_stand/walk_* of 0.82 − 0.07 × 0.92 + 0.33 × 0.36 − 0.08 × 0.92 × 0.36 = 0.85. Entering the values of ILR1 = 1.42 and ILR2 = 0.85 in the equations presented in Supplementary Material File S1, steps C2–4, leads to the predicted percentages of time spent sitting, standing and walking (rounded off to one decimal): 70.5%time, 22.6%time, and 6.8%time.

## 4. Discussion

This is the first calibration study to predict ‘true’ time spent in a complete set of physical behaviors (sitting, standing and walking) during office work on the basis of the self-reports of these behaviors, using a compositional data analysis (CoDA) approach. In summary, we found that un-calibrated compositions of self-reported sitting, standing and walking were inaccurate, particularly at the individual level. Our calibration models, based on self-reported sitting, standing and walking, were effective in increasing the accuracy of estimated behaviors; errors were reduced to between 24% and 40% of those obtained with un-calibrated self-reports. The calibration models remained effective for follow-up data on the same workers. Overall, our findings suggest that calibration using a CoDA approach is a promising tool for improving accuracy in studies based on self-reported physical behaviors.

Workers considerably underestimated lower time proportions and overestimated higher time proportions, expressed in terms of isometric log-ratios (ILR), for sitting, standing and walking (Figure 2), which may reflect both social desirability and recall bias [18,40]. A similar tendency to underestimate “short” and overestimate “long” durations of an activity has been reported for computer work [41], but it does not appear to be a general phenomenon across a variety of occupational tasks [42]. Our results corroborate previous studies reporting both random and systematic errors in self-reported behaviors during office work [18,43,44]. However, we found that calibration was effective at reducing errors in self-reported sitting, standing and walking relative to the other two behaviors, to 36%, 40% and 24% of the error associated with un-calibrated self-reports, respectively. Thus, the calibrated estimates of ‘true’ behaviors, as determined by accelerometry, were considerably closer to that truth than un-calibrated self-reports (Figure 2). Validation of the calibration models using new data from the same workers at three-months follow-up showed similar reductions of errors to those at baseline, indicating that the models were robust. Thus, after appropriately processing time-use data in the CoDA space, our calibration models appear useful for improving the accuracy of self-reported estimates of sitting, standing and walking time during office work in similar populations.

A novelty of this study is the use of the complete composition of multiple self-reported physical behaviors to predict ‘true’ time use. In a previous calibration study in the same population, we focused only on sitting [35] and expressed sitting time using a single ILR (i.e., sit/nonsit) without considering that self-reported information on behaviors occurring during non-sitting (i.e., standing and walking) might be of additional predictive value for sitting. In that study, we found that the simple calibration model reduced RMS error for the sit/nonsit ILR to 55% of the error before calibration. In the current study, we used the complete set of self-reported physical behaviors to predict true sitting, expressed in terms of two ILRs [30], for example, *ILR*1*_sit/nonsit_* and *ILR*2*_stand/walk_* when sitting was the prioritized behavior. We found that both self-reported ILR coordinates as well as their interaction contributed significantly to the prediction of ‘true’ sitting (Table 3). Calibration resulted in an even larger reduction in RMS error, that is, down to 36% of the error before calibration, than that obtained using the simple model in our previous paper. This pronounced ability of ILR2 to improve the accuracy of self-reports is illustrated by the prediction curves in Figure 2. For standing, the second ILR (*ILR*2*_sit/walk_*) contributed less to prediction of ‘true’ behavior, as indicated by the small and borderline significant estimate (Table 3). Still, the prediction curves for standing were steeper with decreasing values of ILR2, as illustrated in Figure 2.

The explained variance for the models predicting sitting (39%), standing (27%), and walking (5%) indicated that self-reports contained more useful information for calibration of sitting and standing compared to walking in the current sample of office workers. Even so, the calibration of walking was very effective in improving accuracy and even outperformed models on sitting and standing in terms of error reduction. This can be explained by the considerable intercept of the calibration model for walking (Table 3) that, together with the almost zero regression slope, shows that the most accurate estimates of ‘true’ walking behavior can be obtained by simply assuming that all individuals behave (almost) as the group mean, with very little additional predictive value of what they say themselves. This limited predictive value of self-reported walking may be explained by difficulties in recalling behaviors like walking that occur rarely and likely in short periods at a time during office work, compared to sitting and standing that occur much more, and for longer uninterrupted periods [18].

While individual self-reported estimates of time spent sitting, standing and walking suffered from notable inaccuracy compared to ‘true’, objectively measured estimates of these behaviors, we found that estimates of the group mean obtained by self-reports were very close to the means of objective measurements. As a tentative interpretation, this may suggest that self-reported behaviors among office workers are useful, even without calibration, in epidemiological studies using a group-based exposure assessment strategy [45], while they are not recommended for studies using an individual-based strategy.

### 4.1. Practical Applications and Generalizability of Calibration Models

One strength of this study is the use of CoDA for calibrating self-reported physical behaviors using the complete composition of physical behaviors at work; that is, sitting, standing and walking. The current findings suggest that our calibration models, developed in a CoDA framework, can be used as a cost-effective way of improving the accuracy of self-reported estimates of physical behaviors among office workers. Similar models can be developed in the context of large-scale cohort or intervention studies. Objective measurements and self-reports may then be collected in a representative sub-sample of participants at baseline, and the resulting calibration model used to calibrate self-reported data in the entire study population across several data collection waves in time. This offers new opportunities in research devoted to understanding the health effects of physical behaviors and it supports effective large-scale surveillance. Moreover, our findings may facilitate professionals in the development of effective policies to improve occupational health and safety for the workers. Appropriate initiatives for changing workers’ physical behaviors, including the eventual evaluation of their effectiveness, obviously must be based on accurate data quantifying those behaviors. Consider, for example, a large organization aiming to implement sit/stand tables to reduce prolonged sitting time in workers. In this case, self-reports will likely be used to evaluate the effectiveness of the intervention, as objective measurements might be too expensive. To offset the inaccuracy of self-reported data and the resulting risk for incorrect conclusions, calibration of these self-reports, either using the equations presented in Supplementary Material File S1 or a population-specific customized set of calibration equations may be an effective action.

Validation of our models using new data from the same population is another study strength, even if it is ideal to have data from a separate study population to assess the generalizability of calibration models to other populations. The study sample represented only one organization in Sweden, and our findings may not extend to other organizations and other countries. The large occurrence of private offices relative to different types of open-plan offices at baseline may also have influenced calibration to some extent. However, we suggest this possible effect to be small, since the calibration was valid at follow-up, even though most workers at that time were located in activity-based offices with open spaces. Accuracy of self-reports can depend on various individual factors, such as age, gender, body mass index, and pain [46], as well as on contextual and individual factors influencing the type and pattern of physical behavior [42]. Previous studies have addressed the contribution of such factors to the accuracy of calibration of self-reported physical behaviors [15,16,25,35]. However, we found in a previous study on the same population that adding a broad range of self-reported factors did not substantially improve prediction of sitting time [35]. Thus, in the present study we decided not to include self-reported predictors beyond physical behaviors. In a practical context, this reflects the situation of self-reported behaviors being the only available information from participants. Future studies should address whether calibration—not only using our models—would also be effective in new samples of office workers, as well as in other occupational groups, with other physical behaviors at work. In addition, as this calibration study only addressed working hours, future studies should also examine physical behaviors during non-work time.

### 4.2. Methodological Discussion

CoDA essentially transforms data so that calibration models can be developed in a space of log-transformed ratios that have the properties required for standard least-square regression [29]. This means that the estimates obtained from the models also pertain to data in the CoDA space (i.e., ILRs), which complicates interpretation of results in real space, such as estimation of the actual %time spent in a specific behavior during a working day. We present the results of our models in ILR coordinates to emphasize that compositional data should be processed and analyzed according to CoDA procedures. However, as explained in the calibration example in Section 3.5, calibrated ILR coordinates can be back-transformed to the standard space to obtain data that are more intuitive and interpretable.

A general challenge when applying CoDA is the inability of log-transformed ratios to accommodate zero minutes in any behavior [32]. In the current study, we replaced the few self-reported zeroes for standing and walking time with a value of 1 min per working day, since standing and walking are not expected to occur for less than 1 min during office work, as supported by the objective measurements in this study. While this kind of replacement procedure is common in CoDA literature, other procedures have also been suggested for handling essential zeros [32], and another choice would likely have led to other calibration models, with the extent of the difference compared to our models depending on the occurrence of zeroes in data. Thus, handling of zeroes in self-reported or measured behaviors deserves more attention in order to eventually develop standardized procedures adapted to physical behaviors.

Self-report and objective measurements were not always collected over the same five-day time period. This may have introduced some disagreement between the two sources of data since behaviors will, to some extent, vary between days and weeks [47]. However, adjusting for the time period (self-report before or after objective measurements) did not have any statistically significant effect on the model estimates (results not shown). Thus, we do not expect disparate time windows of data collection to be of notable concern in our case.

Finally, we used a validated software, Acti4, customized to identify distinct activity types on basis of the output from a thigh-worn Actigraph device [20]. This software generates similar outputs for various tri-axial accelerometers (e.g., Actigraph, ActivPAL and Axivity; Professor Andreas Holtermann, personal communication) and we therefore expect calibration results to differ only marginally between different devices.

## 5. Conclusions

Calibration of self-reported compositions of physical behaviors at work was effective in increasing the accuracy of estimated sitting, standing and walking, and the calibration models remained effective when used on new data from the same workers. Calibration of self-reports may therefore offer a cost-effective method for obtaining physical behavior data with a satisfying accuracy in large-scale cohort and intervention studies.

## Figures and Tables

**Table 1:** Demographic information of the study sample at baseline (*n* = 98).

Variable	*n*	%	Mean	SD
Age (years)	98		47	9
Gender (women)	48	49		
Highest education				
Public school	1	1		
High school	29	30		
Vocational	6	6		
University	62	63		
Managing position	17	17		
Office type				
Private office	58	59		
Shared room/open plan	40	41		
Full-time employment	96	97		
Seniority in the work tasks (years)			5	5
Seniority in the organization (years)			13	11

**Table 2:** Time spent in physical behaviors at work according to self-reports and objective measurements (*n* = 98).

Physical Behavior	% of Total Time at Work		
	Mean	SD	Range
*Self-report*			
Sitting	71.7	20.0	10.0–99.6
Standing	21.2	18.0	0.2–77.8
Walking	7.2	7.3	0.2–50.0
*Objective measurement*			
Sitting	69.8	14.7	25.3–90.7
Standing	23.8	14.4	4.4–70.7
Walking	6.4	2.2	2.5–13.8

**Table 3:** Calibration models predicting ‘true’ time use in physical behaviors at work from the compositions of self-reported behaviors. Models are based on compositional data expressed in isometric log-ratios (ILR).

Self-Reported Predictors	B	SE	*p*	R^2^	RMS before Calibration	RMS after Calibration	% of RMS before Calibration
*Sitting*							
Intercept	1.39	0.10	<0.001	0.39	1.03	0.37	36
ILR1 Sit/nonsit	0.14	0.04	0.001				
ILR2 Stand/walk	−0.39	0.09	<0.001				
Interaction (ILR1 × ILR2)	0.11	0.03	<0.001				
*Standing*							
Intercept	0.24	0.11	0.03	0.27	1.24	0.50	40
ILR1 Stand/nonstand	0.30	0.09	<0.001				
ILR2 Sit/walk	−0.09	0.05	0.06				
Interaction (ILR1 × ILR2)	−0.04	0.04	0.26				
*Walking*							
Intercept	−1.45	0.07	<0.001	0.05	1.21	0.29	24
ILR1 Walk/nonwalk	0.03	0.04	0.37				
ILR2 Sit/stand	0.03	0.03	0.33				
Interaction (ILR1 × ILR2)	0.01	0.02	0.68				

Note: B coefficients are shown for prediction of the objectively measured ILR1 from self-reported ILR1 and ILR2 for the prioritized behavior noted in the first column. The performance of the models is indicated by R^2^ and RMS error before and after calibration; % of RMS is calculated as the RMS error associated with estimates as predicted by the calibration model (Table 3), relative to the RMS error of un-calibrated data (lower values indicate better performance).

## Supplementary Materials

The following are available online at https://www.mdpi.com/1660-4601/16/17/3111/s1, File S1: Procedure for transforming three-part compositions according to CoDA, calibrating self-reported compositions into estimates of ‘true’ values, and back-transforming the resulting estimates to the real space; File S2: Models predicting the second objectively measured isometric log ratio, that is, ILR2, from self-reported ILRs in sets with sitting, standing and walking as the primary variable.
