# Supplementary material for: Calibration of Self-Reported Time Spent Sitting, Standing and Walking among Office Workers: A Compositional Data Analysis
Source: Int J Environ Res Public Health. 2019 Aug 27;16(17):3111. doi: 10.3390/ijerph16173111 (PMC6747301; doi:10.3390/ijerph16173111)
Supplement: Supplementary file 1 [file ijerph-16-03111-s001.zip › File S1.docx]

**Procedure for transforming three-part compositions according to CoDA, calibrating self-reported compositions into estimated ‘true’ values, and back-transforming the resulting estimates to real space^[[1]](#footnote-1)^**

1. **Transformation of compositions in real space to Isometric Log Ratios (ILR)**

A1. Consider a data set containing, from a number of workers, their three-part compositions of self-reported time (%) in activities: A, B, C; and the corresponding compositions objectively measured using accelerometry: A, B, C

A2. Express these compositions in CoDA space, as ILR sets:

*Self-reports:*
ILR1 = SQRT(2/3)*LN(A/SQRT(B*C))
ILR2 = SQRT(1/2)*LN(B/C)

*Objective measurements:*
ILR1 = SQRT(2/3)*LN(A/SQRT(B*C))
ILR2 = SQRT(1/2)*LN(B/C)

1. **Calibration of self-reported Isometric Log Ratios into estimated ‘true’ values**

B1. Resolve regression models expressing ILRs according to objective measurements as a linear function of ILRs according to self-reports:

Model 1: ILR1 = a1 + b1*ILR1 + b2*ILR2 + b3*ILR1*ILR2
Model 2: ILR2 = a2 + b4*ILR1 + b5*ILR2 + b6*ILR1*ILR2

B2. Use the resolved regression models, i.e. the coefficients a1-a2 and b1-b6, to obtain estimates, ILR1 and ILR2, of the ‘true’ ILR coordinates for any particular individual from her self-reported ILR1 and ILR2:

ILR1 = a1 + b1*ILR1 + b2*ILR2 + b3*ILR1*ILR2
ILR2 = a2 + b4*ILR1 + b5*ILR2 + b6*ILR1*ILR2

1. **Back-transformation of estimated ‘true’ ILR values to compositions in real space**

C1. Rearrange the equations expressing the estimated ILR1 and ILR2 in terms of real-space values:

ILR1 = SQRT(2/3)*LN(A/SQRT(B*C))
This is equivalent to: A/SQRT(B*C) = EXP(SQRT(3/2)*ILR1)

ILR2 = SQRT(1/2)*ln(B/C).
This is equivalent to: B/C = EXP(SQRT(2)*ILR2)

C2. Set (for the ease of reading):

EXP(SQRT(3/2)*ILR1) = Q
EXP(SQRT(2)*ILR2) = R

C3. Now, three equations with three unknowns are available to obtain A, B, C, i.e. estimates of %time in the three parts of the composition, expressed in real space:

A/SQRT(B*C) = Q
B/C = R
A+B+C = 100

C4. Rearranging these equations gives the solutions for estimated values of A, B, C (%) in real space:

A = 100*Q*SQRT(R)/(1+R+Q*SQRT(R))
B = 100*R/(1+R+Q*SQRT(R))
C = 100/(1+R+Q*SQRT(R))

1. Colour code: red, self-reports; green, objective measurements; violet, estimates of objectively measured values, obtained by calibration of self-reports [↑](#footnote-ref-1)
