# Supplementary material for: Calibration of Self-Reported Time Spent Sitting, Standing and Walking among Office Workers: A Compositional Data Analysis
Source: Int J Environ Res Public Health. 2019 Aug 27;16(17):3111. doi: 10.3390/ijerph16173111 (PMC6747301; doi:10.3390/ijerph16173111)
Supplement: Supplementary file 1 [file ijerph-16-03111-s001.zip › File S2.docx]

**Table.** Models predicting the second objectively measured isometric log ratio, i.e. ILR2, from self-reported ILRs in sets with sitting, standing and walking as the primary variable.

|  | **B** | **SE** | **p** | **R^2^** |
| --- | --- | --- | --- | --- |
| ***Sitting (prediction of ILR2: stand/walk)*** |  |  |  |  |
| Intercept | 0.82 | 0.11 | <0.01 | 0.24 |
| ILR1 Sit/non-sit | -0.07 | 0.04 | 0.12 |  |
| ILR2 Stand/walk | 0.33 | 0.10 | <0.01 |  |
| Interaction (ILR1 x ILR2) | -0.08 | 0.03 | 0.02 |  |
| ***Standing (prediction of ILR2: sit/walk)*** |  |  |  |  |
| Intercept | 1.50 | 0.06 | <0.01 | 0.19 |
| ILR1 Stand/non-stand | -0.07 | 0.05 | 0.14 |  |
| ILR2 Sit/walk | 0.09 | 0.03 | <0.01 |  |
| Interaction (ILR1 x ILR2) | 0.00 | 0.02 | 0.98 |  |
| ***Walking (prediction of ILR2: sit/stand)*** |  |  |  |  |
| Intercept | 0.63 | 0.12 | <0.01 | 0.33 |
| ILR1 Walk/non-walk | 0.07 | 0.06 | 0.22 |  |
| ILR2 Sit/stand | 0.21 | 0.05 | <0.01 |  |
| Interaction (ILR1 x ILR2) | -0.06 | 0.03 | 0.05 |  |
